# Teamwork for smoking cessation: which smoker was willing to engage their partner? Results from a cross-sectional study

**DOI:** 10.1186/s13104-020-05183-2

**Published:** 2020-07-20

**Authors:** Catherine S. Nagawa, Oluwabunmi M. Emidio, Kate L. Lapane, Thomas K. Houston, Bruce A. Barton, Jamie M. Faro, Amanda C. Blok, Elizabeth A. Orvek, Sarah L. Cutrona, Bridget M. Smith, Jeroan J. Allison, Rajani S. Sadasivam

**Affiliations:** 1 Department of Population and Quantitative Health Sciences, University of Massachusetts Medical School, 368 Plantation Street, Worcester, MA 01605 USA; 2 Veterans Affairs Center for Clinical Management Research, Veterans Affairs Ann Arbor Healthcare System, United States Department of Veterans Affairs, Ann Arbor, MI USA; 3 Systems, Populations and Leadership Department, School of Nursing, University of Michigan, Ann Arbor, MI USA; 4 Learning Health Systems, Department of Internal Medicine, Wake Forest University School of Medicine, Winston-Salem, NC USA; 5 Center for Healthcare Organization and Implementation Research, Bedford VA Medical Center, Bedford, MA USA; 6 Center of Innovation for Complex Chronic Healthcare, Spinal Cord Injury Quality Enhancement Research Initiative, Hines VA Medical Center, Chicago, IL USA; 7 Department of Pediatrics, Feinberg School of Medicine, Northwestern University, Evanston, IL USA

**Keywords:** Partner support, Smoking cessation, Seeking support, Willingness, Partner engagement

## Abstract

**Objective:**

Smokers are greatly influenced by those living with them, but strategies that increase partner support for smoking cessation are lacking. Using a cross-sectional study design, we explored factors associated with willingness to engage a partner in smoking cessation in smokers registered on a web-assisted tobacco intervention trial.

**Results:**

Study participants (n = 983) were recruited between July 2018 and March 2019. About 28% of smokers were willing to engage their partner in cessation efforts. The odds of willingness to engage a partner were more than two-fold for smokers reporting presence of other smokers in the immediate family (adjusted odds ratio (aOR): 2.18; 95% confidence interval (CI) 1.51–3.15 for 1–3 smokers; aOR, 3.12; 95% CI 1.95–4.98 for ≥ 4 smokers) compared to those with no smokers in the immediate family. Women had lower odds of willingness to engage (aOR; 0.82; 95% CI 0.58–1.16) than men, but this was not statistically significant. Use of e-cigarettes and visitation to a smoking cessation website prior to the intervention were both positively associated with willingness to engage partners in cessation. Future research should assess whether interventions tailored to smokers willing to engage partners or spouses could increase effectiveness of partner support during cessation.

## Introduction

Smokers are greatly influenced by those living with them [1–4]. Among US adults, the rates of smoking among married individuals or those living with a partner (13%) is less than the rate of smoking in single/never married (14%), and divorced (18%) individuals [5]. Smokers who frequently receive positive support from spouses or partners are more likely to successfully quit [3, 6]. Further, incorporating strategies such as implementing a smoke-free home strongly promotes quitting and prevents relapse [7]. Developing behavioral interventions to increase partner support for smoking cessation is therefore an important research goal.

Past smoking cessation studies that aimed to evaluate effectiveness of partner support on long-term cessation were evaluated in a recent Cochrane review. [13] Findings from the Cochrane review indicated that partner support did not significantly impact long-term smoking cessation. [13] The review broadly defined partners as spouses, friends, relatives, co‐workers, or fellow cessation participants. Studies in which spouses or intimate partners were enlisted as support partners reported larger differences in quit rates between smokers who received and those who did not receive support [8, 9]. This indiates that spouses or intimate partner support may have an impact on long-term cessation. But since most trials did not increase partner support, this review recommended additional research on ways to increase partner support for cessation before evaluating effectiveness of partner support on long-term cessation.

Understanding which smokers to target for partner support smoking cessations interventions is key to designing effective interventions that increase chances of long-term smoking cessation. Research suggests that individuals who seek out social support are more likely to value the support they receive [10], and may be ideal targets for these interventions. Therefore, we sought to identify demographic factors and smoking behaviors associated with a smokers’ willingness to engage their partners or spouses in smoking cessation interventions.

## Main text

### Study design

We conducted a cross-sectional study design using baseline data collected in a web-assisted tobacco intervention trial. Details about the web-assisted tobacco intervention have been published [11]. This study was approved by the Institutional Review Board at the University of Massachusetts Medical School.

### Study setting and recruitment

Study participants (n = 983) were current smokers, aged 18 years or older, able to read and speak English, and had internet access at home. Between July 2018 and March 2019, participants were recruited using Google and Facebook advertisements, and through ResearchMatch (a free and secure online tool that allows people in the United States to create a profile to match them to research projects) [17]. ResearchMatch volunteers who expressed interested in participating in our study received an email which described the study in detail and contained a link to the study website.

### Measures

#### Willingness to engage a partner

To assess willingness to engage a partner, we used the question, “Would you be interested in a program that also includes your partner or spouse in smoking cessation?” Smokers indicated their willingness to engage a partner with a yes or no response. Because we did not provide a ‘not applicable’ option, participants who responded ‘no’ were used as a reference category and included; (1) smokers unwilling to engage a partner, and (2) those without partners/spouses.

#### Demographic and smoking-related factors

Demographic characteristics assessed included gender, age, education, and race/ethnicity. We collected data on cigarettes per day, readiness to quit was based on stages of change, [12] nicotine dependence using the Fagerstrom test [13], and e-cigarette use. Having other smokers in the immediate family was categorized as having; no smokers, 1–3 smokers, and ≥ 4 smokers. Separate questions regarding use of Nicotine Replacement Therapy (NRT), visiting a cessation website, and tobacco counselling were included with yes/no response options.

#### Statistical analysis

We calculated the percent distributions or mean with standard deviations (SD) for demographic factors, smoking-related factors by willingness to engage a partner. We conducted logistic regression analysis using willingness to engage a partner as the dependent variable and using demographic and smoking-related factors as independent variables to estimate unadjusted odds ratios (ORs), adjusted ORs (aOR) and 95% confidence intervals (95% CI). To assess multicollinearity, we calculated Cramer’s V or phi statistic to measure the strength of associations between smoking-related variables. Coefficients greater than 0.5 indicated a strong association [14, 15], and may signal that two variables measure similar concepts. We also fit a multivariable linear regression model to calculate the variance inflation factor (VIF) and tolerance. Variables with VIF greater than 10 indicate presence of multicollinearity. Model specification was assessed using the Hosmer–Lemeshow goodness of fit test for logistic regression [16]. All analyses were conducted with Stata software (v.15) in 2019.

### Results

Majority of the participants were women (74%), 36% were 55 years and older, and 30% between 19 to 34 years old. Most either had some college education or were college graduates (69%), had used e-cigarettes (78%), and 64% reported presence of other smokers in the immediate family. Less than half used NRT (47%) or tobacco counselling (21%) for quitting.

Table 1 shows that 27.7% were willing to engage a partner in smoking cessation efforts. Demographic factors were balanced between smokers willing and those unwilling to engage a partner except for age. Twenty-five percent of participants were willing to engage a partner in cessation efforts, and 41% of those unwilling were ≥ 55 years of age. Smokers willing to engage a partner, on average, smoked 17.6 cigarettes per day (SD, 9.6) and those unwilling an average 16.3 cigarettes per day (SD, 8.7). Seventy-eight percent of those willing and 78% of those willing to engage a partner reported at least one smoker in the immediate family. Regardless of willingness to engage a partner, 47% had tried NRT.

**Table 1:** Frequencies and percent distributions of demographic and smoker’s characteristics by Willingness to engage of partners in smoking cessation among smokers who participated in a web-assisted tobacco intervention trial (N = 983)

Demographic factors	N = 983 n (%)	Willing to engage partner in smoking cessation	
		Yes (n = 272)	No (n = 711)
Demographics		*Column percentage (%)*	
Gender			
Women	731 (74%)	71	76
Age group (years)			
19–34	291 (30%)	38	27
35–44	192 (20%)	23	18
45–54	141 (14%)	15	14
55+	359 (36%)	25	41
Education^b^			
High school	297 (30%)	36	28
College	681 (70%)	64	72
Race/ethnicity^b^			
Non-Hispanic white	679 (72%)	71	72
Non-Hispanic black	127 (13%)	15	13
Hispanic	63 (7%)	8	6
Other^a^	81 (9%)	6	9
Smoking-related factors			
Cigarettes/day mean (SD)		17.6 (9.6)	16.3 (8.7)
Readiness for change			
Pre-contemplation	40 (4%)	6	3
Contemplation	561 (57%)	59	56
Preparation	64 (7%)	5	7
Action	243 (25%)	24	25
Maintenance	69 (7%)	7	8
How soon after you wake up do you smoke your first cigarette?			
Within 5 min	385 (39%)	40	39
6–30 min	370 (38%)	37	37
31 to 60 min	111 (11%)	13	11
After 60 min	117 (12%)	10	13
Smokers in immediate family besides self			
No smokers	347 (35%)	22	43
1 to 3 smokers	478 (49%)	61	48
≥ 4 smokers	158 (16%)	17	9
Have you ever tried using e-cigarettes?	763 (78%)	85	75
Participated in tobacco counselling	203 (21%)	28	18
Used Nicotine replacement therapy	464 (47%)	47	47
Visited a smoking cessation website prior to the intervention	445 (45%)	52	43

^a^Other includes Asian, Native Hawaiian, American Indians, Alaska Natives and those not sure about race ^b^missing data: education level, n = 5; readiness level n = 6; Race/ethnicity, n = 32

In the present analysis, we did not detect presence of multicollinearity. Therefore, all variables were included in the logistic model. In the unadjusted and adjusted analyses (Table 2), the odds of willingness to engage a partner tended to increase with decreased age (aOR_45–54 versus 55+ years_: 1.43; 95% CI 0.88–2.32; aOR_35-45 versus 55+_: 1.60; 95% CI 1.03–2.51; aOR_19–34 versus 55+_: 1.87; 95% CI 1.24–2.84). Compared to men, women had lower odds of willingness to engage a partner (aOR; 0.82; 95% CI 0.58–1.16). In the adjusted analysis, smokers who reported e-cigarette use were statistically significantly more likely to report willingness to engage a partner in cessation efforts (aOR: 1.51; 95% CI 1.02–2.25) compared to smokers who did not report e-cigarette use. Compared to having no smokers, the adjusted odds of willingness to engage a partner for smokers who had 1-3 smokers in the immediate family was 2.18 (95% CI 1.51–3.15) and for those with ≥ 4 smokers were 3.12 (95% CI 1.95–4.98). This association statistically significant. Visitation to a smoking cessation website prior to the intervention was statistically significantly associated with greater odds of willing to engage partners (aOR; 1.62; 95% CI 1.18–2.21).

**Table 2:** Odds Ratios and 95% confidence intervals of willingness to engage a partner in smoking cessation among smokers who participated in a web-assisted tobacco intervention trial (N = 983)

Demographic factors	N = 983 n (%)	Unadjusted Odds Ratio (95% confidence interval)	*p* value	Adjusted Odds Ratio (95% confidence interval)	p-value
Age group (years)					
55+	359 (36%)	Reference		Reference	
45–54	141 (14%)	1.73 (1.09–2.71)	0.18	1.43 (0.88–2.32)	0.15
35–44	192 (20%)	2.12 (1.42–3.18)	< 0.01	1.60 (1.03–2.51)	0.04
19–34	291 (30%)	2.35 (1.64–3.37)	< 0.01	1.87 (1.24–2.84)	< 0.01
Gender					
Male	252 (26%)	Reference		Reference	
Women	731 (74%)	0.78 (0.57–1.07)	0.13	0.82 (0.58–1.16)	0.20
Education					
College graduate	681 (70%)	Reference		Reference	
High school	291 (30%)	1.41 (1.04–1.90)	0.02	1.19 (0.86–1.66)	0.33
Race/ethnicity					
Non-Hispanic white	679 (72%)	Reference		Reference	
Non-Hispanic black	127 (13%)	1.20 (0.80–1.81)	0.38	1.12 (0.72–1.75)	0.73
Hispanic	63 (7%)	1.30 (0.75–2.26)	0.34	1.11 (0.62–1.99)	0.78
Other^a^	81 (9%)	0.69 (0.36–1.21)	0.63	0.78 (0.43–1.41)	0.87
Smoking-related factors					
Cigarette packs per day					
Less than 1 pack/day	551 (56%)	Reference	0.61	Reference	0.58
1 or more packs/day	432 (44%)	1.07 (0.81–1.42)		1.10 (0.79–1.55)	
Readiness for change					
Pre-contemplation	40 (4%)	Reference		Reference	
Contemplation	561 (57%)	0.53 (0.28–1.04)	0.06	0.55 (0.27–1.11)	0.12
Preparation	64 (7%)	0.34 (0.14–0.83)	0.02	0.35 (0.14–0.91)	0.03
Action	243 (25%)	0.49 (0.25–0.98)	0.04	0.47 (0.23–1.00)	0.05
Maintenance	69 (7%)	0.37 (0.16–0.89)	0.02	0.30 (0.12–0.76)	0.02
How soon after you wake up do you smoke your first cigarette?					
Within 5 min	385 (39%)	Reference		Reference	
6–30 min	370 (38%)	1.00 (0.73–1.38)	0.99	0.99 (0.70–1.42)	0.96
31 to 60 min	111 (11%)	1.19 (0.76–1.89)	0.44	1.21 (0.73–2.00)	0.52
After 60 min	117 (12%)	0.78 (0.48–1.27)	0.31	0.80 (0.46–1.39)	0.43
Have you ever tried using e-cigarettes?^b^	763 (78%)	1.89 (1.30–2.75)		1.51 (1.02–2.25)	0.04
Smokers in immediate family besides self:					
No smokers	347 (35%)	Reference		Reference	
1 to 3 smokers	478 (49%)	2.61 (1.84–3.71)	< 0.01	2.18 (1.51–3.15)	< 0.01
≥ 4 smokers	158 (16%	3.98 (2.59–6.12)	< 0.01	3.12 (1.95–4.98)	< 0.01
Participated in tobacco counselling^b^	203 (21%)	1.78 (1.29–2.47)	< 0.01	1.47 (0.92–2.37)	0.11
Used nicotine replacement therapy^b^	464 (47%)	1.01 (0.76–1.34)	0.93	0.79 (0.53–1.19)	0.26
Visited a smoking cessation website prior to the intervention^b^	445 (45%)	1.47 (1.11–1.95)	< 0.01	1.62 (1.18–2.21)	< 0.05

^a^Other includes Asian, Native Hawaiian, American Indians, Alaska; ^b^Reference category (no); Hosmer–Lemeshow goodness-of-fit test of adjusted model: p-value > 0.05

Table 3 shows results stratified by gender; 26.4% of women and 31.3% of men were willing to engage a partner in smoking cessation efforts. Men differed from women regarding factors associated with willingness to engage a partner. An association between younger age and willingness to engage a partner in smoking cessation efforts was observed in women, but not men. The inverse association between readiness to quit and willingness to engage a partner in smoking cessation efforts was statistically significant in men, but not women. Factors statistically significantly associated with willingness to engage a partner in men, but not women were prior participation in tobacco counselling (aOR _men_: 2.73 (95% CI 1.01–7.41); aOR _women_: 1.19 (95% CI 0.67–2.12)) and trying e-cigarettes (aOR _men_: 4.41 (95% CI 1.69–11.49); aOR _women_: 1.17 (95% CI 0.74–1.83)).

**Table 3:** Odds ratios and 95% confidence intervals of willingness to engage a partner in smoking cessation among smokers who participated in a web-assisted tobacco intervention trial, stratified by gender (N = 983)

	n (%)	Adjusted Odds Ratio (95% confidence interval) Women (n = 731)	p-value	n (%)	Adjusted Odds Ratio (95% confidence interval) Men (n = 252)	p-value
Demographic factors						
Age group (years)						
55+	289 (40%)	Reference		70 (29%)	Reference	
45–54	102 (14%)	1.40 (0.78–2.51)	0.25	39 (16%)	1.04 (0.38–2.87)	0.87
35–44	137 (19%)	2.15 (1.28–3.62)	< 0.01	55(22%)	0.67 (0.25–1.76)	0.44
19–34	203 (28%)	2.30 (1.41–3.77)	< 0.01	88 (35%)	1.06 (0.44–2.59)	067
Education						
College graduate	507 (70%)	Reference		174 (70%)	Reference	
High school	221 (30%)	1.26 (0.85–1.87)	0.21	76 (30%)	1.31 (0.65–2.63)	0.57
Race/ethnicity						
Non-Hispanic white	524 (75%)	Reference		155 (64%)	Reference	
Non-Hispanic black	88 (12%)	1.38 (0.81–2.33)	0.24	39 (16%)	0.53 (0.21–1.34)	0.19
Hispanic	40 (6%)	1.89 (0.92–3.87)	0.08	23 (10%)	0.29 (0.09–0.97)	0.05
Other^a^	51 (7%)	0.82 (0.40–1.73)	0.95	30 (10%)	0.57 (0.18–1.74)	0.85
Smoking-related factors						
Cigarette packs per day						
Less than 1 pack/day	423 (58%)	Reference		128 (51%)	Reference	
1–2 or more packs/day	308 (42%)	1.16 (0.77–1.74)	0.49	124 (49%)	1.08 (0.56–2.09)	0.93
Readiness for change						
Pre-contemplation	30 (4%)	Reference		10 (4%)	Reference	
Contemplation	417 (58%)	0.75 (0.33–1.75)	0.55	144 (57%)	0.23 (0.08–0.99)	0.06
Preparation	39 (5%)	0.64 (0.21–1.98)	0.47	25 (10%)	0.07 (0.01–0.48)	< 0.01
Action	189 (26%)	0.70 (0.30–1.70)	0.45	54 (21%)	0.17 (0.03–0.87)	0.03
Maintenance	50 (7%)	0.34 (0.11–1.06)	0.09	19 (8%)	0.18 (0.12–0.76)	0.09
How soon after you wake up do you smoke your first cigarette?						
Within 5 min	291 (40%)	Reference		94 (37%)	Reference	
6–30 min	275 (37%)	0.96 (0.63–1.46)	0.89	95 (38%)	0.98 (0.48–2.01)	0.94
31 to 60 min	78 (11%)	1.10 (0.60–2.05)	0.77	33 (13%)	1.33 (0.49–3.58)	0.64
After 60 min	87 (12%)	0.82 (0.43–1.59)	0.60	30 (12%)	0.75 (0.25–2.28)	0.71
Have you ever tried using e-cigarettes?^b^	566 (77%)	1.17 (0.74–1.83)		197 (78%)	4.41 (1.69–11.49)	< 0.01
Smokers in immediate family besides self						
No smokers	247 (34%)	Reference		100 (40%)	Reference	
1 to 3 smokers	369 (51%)	2.08 (1.34–3.24)	< 0.01	109 (43%)	2.43 (1.15–3.15)	0.02
≥ 4 smokers	115 (16%)	3.08 (1.80–5.37)	< 0.01	43 (17%)	3.88 (1.51–9.94)	< 0.01
Participated in tobacco counselling^b^	124 (17%)	1.19 (0.67–2.12)	0.50	79 (31%)	2.73 (1.01–7.41)	0.06
Used nicotine replacement therapy^b^	333 (46%)	0.85 (0.54–1.34)	0.50	131 (52%)	0.57 (0.22–1.45)	0.22
Visited a smoking cessation website prior to the intervention^b^	332 (45%)	1.72 (1.18–2.50)	< 0.01	113 (45%)	1.31 (0.67–2.53)	0.45

^a^Other includes Asian, Native Hawaiian, American Indians, Alaska; ^b^Reference category (no)

### Discussion

Over one quarter of smokers were willing to engage a partner in smoking cessation efforts. Individuals with other smokers in their immediate family, smokers who had previously visited a smoking cessation website, tried using e-cigarettes, or had a lower education level were more likely to report willingness to engage a partner, while women and older smokers were less likely to report such willingness.

Our findings are consistent with Carlson et al. [17], who reported that 26% of smokers encouraged to bring a support person to a smoking cessation program did so. Greaney et al. [18] reported higher proportions (50%) of smokers willing to engage partners, though this study targeted multiple health behavioral changes (physical activity, fruit and vegetable intake, red meat consumption, multivitamin use, and smoking) whereas our study targeted only smoking cessation. The proportion of smokers willing to engage partners or spouses in cessation represents a target population for whom partner support interventions may be particularly beneficial.

In general, women tended to be less likely to report willingness to engage a partner. Past research shows that the type of support (emotional versus instrumental) required, moderates when men and women are effective in providing social support [19], which may affect support seeking. Female smokers attempting to quit anticipate receiving less support from their spouses [17], and this may explain why women are less willing to engage partners in cessation efforts. Interventions designed to enhance partner support could benefit from understanding how gender roles, as determined by social norms, affect expectations in the context of social support.

Presence of smokers in one’s immediate family was associated with greater odds of willingness to engage a partner. Quitting is more difficult when one’s partner continues to smoke [1, 20] and evidence shows that smokers in dual-smoker relationships express a desire for the partner’s support when quit smoking [20]. Previous research shows that when one spouse stops smoking the other spouse is 67% less likely to smoke [21]. Perhaps creating a ‘we” mindset amongst couples, families or households may be a more effective strategy for quitting than focusing solely on the individual smoker. This approach for behavior change is grounded in family systems and interpersonal theories [22–26], and has been shown to be effective for improving diabetes self-care among adults [27].

We found that smokers who reported e-cigarette use were also more likely to report willingness to engage a partner. Although quality of evidence is low [28], e-cigarettes are frequently marketed as healthier than regular cigarettes [29]. As such, smokers who use e-cigarettes are perhaps actively seeking or testing various cessation strategies. This could explain the observed positive association between e-cigarettes use and increase in willing to engage partners. Given the uncertainities surrounding e-cigarette use, an in-depth exploration of reasons for e-cigarettes use is needed to clarify our finding.

Visiting a smoking cessation website prior to our intervention was predictive of one’s willingness to engage a partner in cessation efforts. This suggests that individuals interested in online cessation programs may also wish to engage a support partner. Recent systematic reviews present evidence for effectiveness of tailored online interventions on quit rates among adults [30–32], but detect no additional benefit from support provided by nurses, pharmacist, coaches or tobacco treatment specialists [30]. There is suggestion that online support such as receiving Facebook likes predicts smoking reduction [33]. One potential area for online interventions is to explore if use of a partner or spouse as a support source, in addition to the various components of the online interventions, provides added benefits to quitting. Receiving support from partners/spouses is cost free and thus more sustainable for long-term cessation.

An exploration of factors associated with a smoker’s willingness to engage a partner in smoking cessation efforts provides insights for future cessation interventions. Interventions tailored to a smoker’s needs for support are more likely to achieve the goal of increasing partner support. In view of prior research, other factors such as partner’s willingness to provide support should also be considered [34].

### Conclusion

We observed characteristic differences between smokers who were willing to engage their partner in smoking cessation efforts and those who were not willing. We recommend designing partner support interventions that target smokers who are willing to engage their partners in cessation to appropriately evaluate the effectiveness of partner support on long-term cessation. Thus, future research can assess whether interventions tailored to the smoker’s need for partner support provide evidence for its effectiveness on long-term smoking abstinence.

## Limitations

- The proportion of participants willing to engage a partner in cessation may have been underestimated in our study. The denominator value used in calculating the proportion of participants willing to engage a partner included those without a partner/spouse, since we did not provide a ‘not applicable’ option to exclude such smokers.
- Our study participants were mostly women, college graduates, and identified as non-Hispanic white race. Generalizability of our study findings is limited as observed associations may manifest differently in other subpopulations.

## Data Availability

Data is available on reasonable requests.

## Abbreviations

aOR: Adjusted Odds Ratio
95% CI: Confidence interval
NRT: Nicotine replacement therapy
SD: Standard deviations
VIF: Variance inflation factor
e-cigarettes: Electronic cigarettes
